# Correction: Yamada et al. Extended-Synaptotagmin 1 Enhances Liver Cancer Progression Mediated by the Unconventional Secretion of Cytosolic Proteins. *Molecules* 2023, *28*, 4033

**DOI:** 10.3390/molecules31050777

**Published:** 2026-02-26

**Authors:** Kohji Yamada, Yoshito Hannya, Tsunekazu Oikawa, Ayano Yoshida, Kuniko Katagiri, Saishu Yoshida, Rei Koizumi, Naoko Tago, Yuya Shimoyama, Akira Kawamura, Yuta Mochimaru, Ken Eto, Kiyotsugu Yoshida

**Affiliations:** 1 Department of Biochemistry, The Jikei University School of Medicine, 3-25-8 Nishi-Shinbashi, Minato-ku, Tokyo 105-8461, Japan; 2 Department of Surgery, The Jikei University School of Medicine, 3-25-8 Nishi-Shinbashi, Minato-ku, Tokyo 105-8461, Japan; 3 Division of Gastroenterology and Hepatology, Department of Internal Medicine, The Jikei University School of Medicine, 3-25-8 Nishi-Shinbashi, Minato-ku, Tokyo 105-8461, Japan

Following publication, concerns were raised regarding the peer-review process related to the publication of this article [1]. Adhering to our standard procedure, the Editorial Board conducted an investigation which determined that, while the peer-review process did comply with MDPI’s Editorial process policy (https://www.mdpi.com/editorial_process), the contribution of one of the two reviewers did not comply with MDPI’s Guidelines for Reviewers (https://www.mdpi.com/reviewers#_bookmark11) and the expectations of the Editorial Board. As a result, the Editorial Board has decided to remove the contribution of one of the reviewers from the open peer-review record (https://www.mdpi.com/1420-3049/28/10/4033/review_report) and conduct a post-publication peer-review to add a new review report.

Based on the new review report, the authors have made the following revisions to this article [1]:

## Figures Correction

Figures 1–5 were replaced by the higher-quality figures in the original publication [1].

## Text Correction

The fifth sentence in Section 2.4 has been changed to “The levels of E-Syt1 expression correlated with a poorer prognosis in grade I (hazard ratio = 3.54, *p* = 0.0087) or grade I + II HCC (hazard ratio = 2.38, *p* = 0.017) (Figure 5C), indicating that E-Syt1 expression effected on liver cancer.”

The Data Availability Statement has been changed as follows.

**Data Availability Statement:** The original contributions presented in this study are included in the article/Supplementary Material. Further inquiries can be directed to the corresponding authors.

The authors state that the scientific conclusions are unaffected. This correction was approved by the Academic Editor. The original publication has also been updated.
