# Supplementary material for: Extended-Synaptotagmin 1 Enhances Liver Cancer Progression Mediated by the Unconventional Secretion of Cytosolic Proteins
Source: Molecules. 2023 May 11;28(10):4033. doi: 10.3390/molecules28104033 (PMC10221710; doi:10.3390/molecules28104033)
Supplement: Supplementary file 1 [file molecules-28-04033-s001.zip › molecules-2395672-supplementary.pdf]

Supplementary Information

Extended-synaptotagmin 1 enhances liver cancer progression mediated by cancer-related unconventional protein secretion

Authors

Kohji Yamada[1,†,*], Yoshito Hannya[1,3,†], Tsunekazu Oikawa[2], Ayano Yoshida[1], Kuniko Katagiri[1], Saishu Yoshida[1], Rei Koizumi[1], Naoko Tago[1], Yuya Shimoyama[1,3], Akira Kawamura[1], Yuta Mochimaru[1], Ken Eto[3], Kiyotsugu Yoshida[1,*]

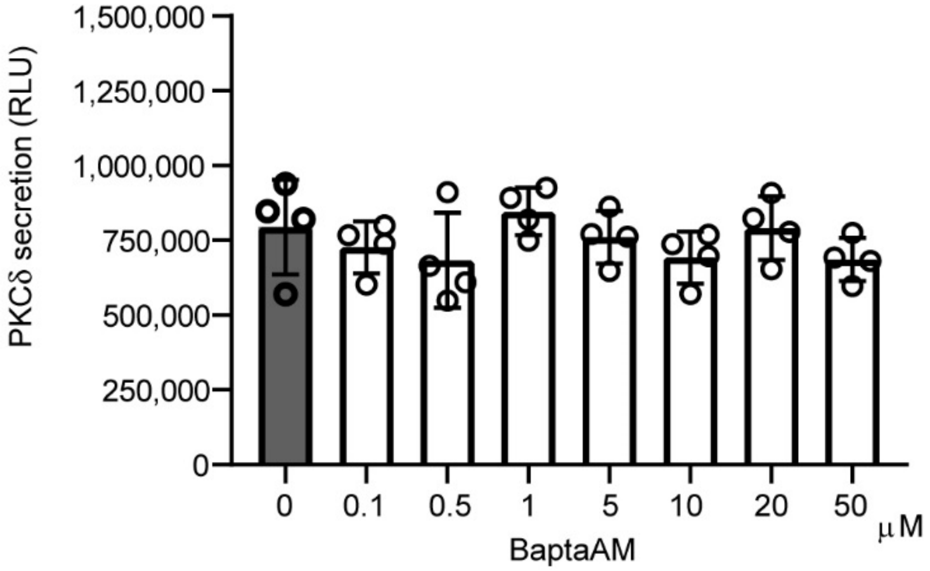

Figure S1. No effect of BaptaAM on PKCδ secretion.

PKCδ secretion measured by HiBiT extracellular assay in doxycycline-inducible HepG2 cells treated with or without BaptaAM at the indicated concentrations for 4 h; *n* = 4 independent experiments. Cells were pre-cultured with a medium containing 0.5 μg / mL doxycycline for 24 h. Data are shown as mean ± s.d.

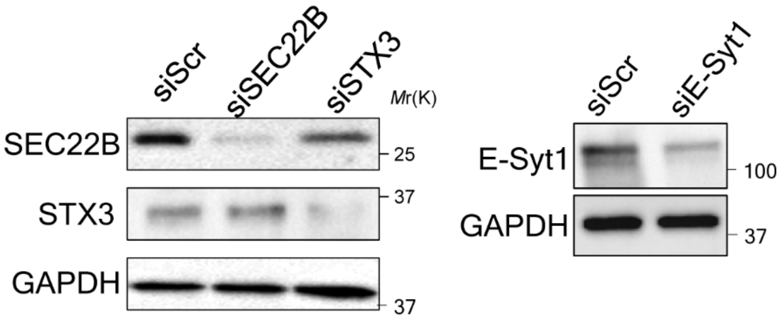

Figure S2. Knockdown in HepG2 cells

Immunoblot analysis of lysates from HepG2 cells treated with scrambled (Scr) or SEC22B, STX3 siRNAs, or E-Syt1 (2 nM) for 48 h. Representative images are shown more than three individual experiments. GAPDH was used as loading control.

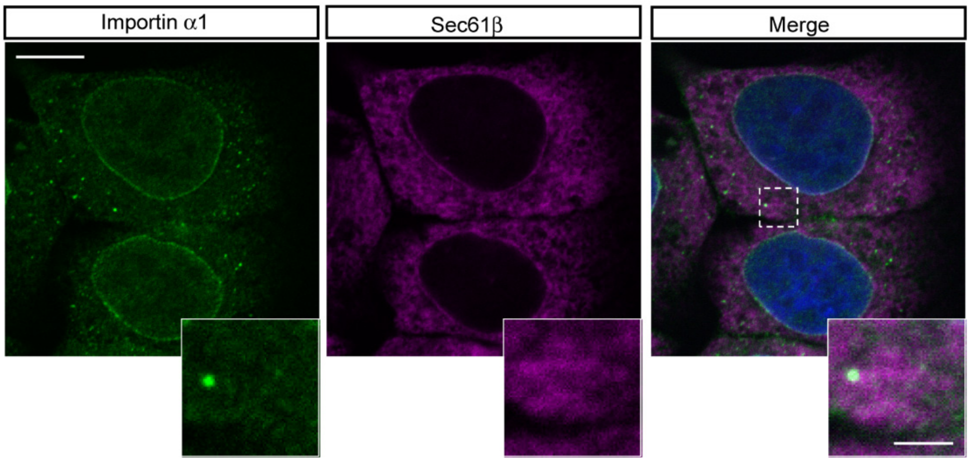

Figure S3. ER localization of importin α1 in HepG2 cells.

Confocal micrographs to detect co-localization of importin α1 on the ER in HepG2 cells. Sec61β and DAPI was used as an ER or nuclear markers, representatively. Scale bar, 10 μm; inset, 2 μm.

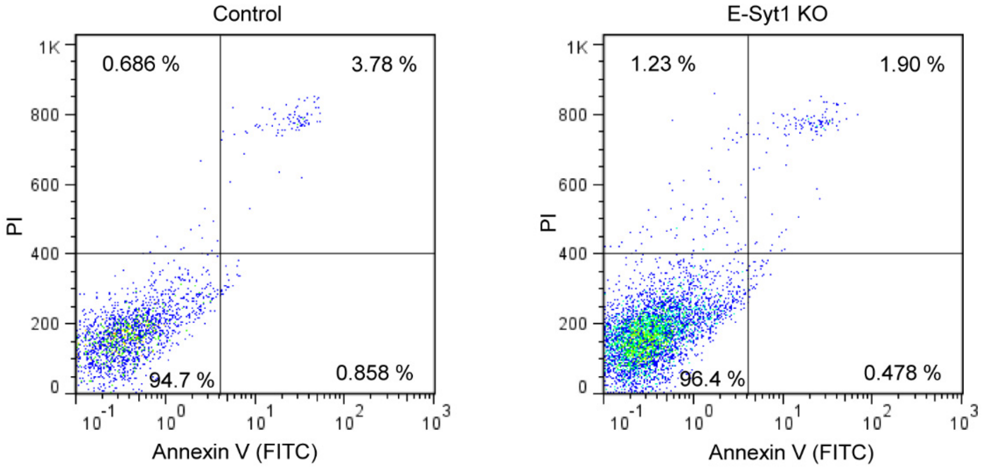

Figure S4. Knockout of E-Syt1 shows no effect on cell apoptosis by serum starvation.

Flow cytometric analysis of control or E-Syt1 KO HepG2 cells incubated under a 0.1% FBS-containing culture condition for 24 h to show independence of E-Syt1 expression in cell apoptosis. Annexin V-PI assay was performed using the Apoptosis Detection kit (Nacalai). Representative data are shown from three individual experiments.
